# Cell Supported Single Membrane Technique for the Treatment of Large Bone Defects: Depletion of CD8^+^ Cells Enhances Bone Healing Mechanisms During the Early Bone Healing Phase

**DOI:** 10.3390/cells15030215

**Published:** 2026-01-23

**Authors:** Marissa Penna-Martinez, Lia Klausner, Andreas Kammerer, Minhong Wang, Alexander Schaible, René Danilo Verboket, Christoph Nau, Ingo Marzi, Dirk Henrich

**Affiliations:** Department of Trauma Surgery and Orthopedics, University Hospital, Goethe University Frankfurt, 60590 Frankfurt am Main, Germany; lia.klausner@stud.uni-frankfurt.de (L.K.); andreas.kammerer@varisano.com (A.K.); m.wang@med.uni-frankfurt.de (M.W.); alexander.schaible@unimedizin-ffm.de (A.S.); verboket@med.uni-frankfurt.de (R.D.V.); c.nau@med.uni-frankfurt.de (C.N.); marzi@trauma.uni-frankfurt.de (I.M.); d.henrich@trauma.uni-frankfurt.de (D.H.)

**Keywords:** bone marrow mononuclear cells, BMC, CD8 lymphocytes, monocytes, mesenchymal stem cells, large bone defect, early bone healing, acellular dermis

## Abstract

**Introduction:** The one-step membrane technique, derived from the Masquelet induced membrane technique, uses human acellular dermal matrix (hADM) that is wrapped around the bone defect to bypass membrane induction, reducing treatment time. Pre-colonization of hADM with bone marrow cells (BMC), particularly after CD8^+^ T cell depletion, enhances bone regeneration. This study examined how CD8^+^ T cell depletion alters the proteins accumulated in the hADM during early healing. **Materials and Methods:** Eighteen male Sprague-Dawley rats received 5 mm femoral defects filled with autologous bone chips and wrapped with hADM, hADM + BMC, or hADM + BMC-CD8. hADMs were recovered on days 3 and 7 (*n* = 3/group/timepoint), incubated ex vivo, and conditioned medium analyzed with a proteome profiler detecting 79 proteins. **Results:** The protein content of the hADM evolved dynamically. At day three, 41 proteins were detected, rising to 47 by day seven, with RGM-A, osteoprotegerin, LIF, IL-6, CCL20, and CCL17 emerging late, consistent with increased regenerative activity. CD8^+^ T cell depletion suppressed early inflammatory and pro-osteogenic mediators (e.g., CCL2, IGF-I, IL-1RA) while upregulating LIX. By day seven, regenerative mediators (CCL20, GDF-15, RGM-A) were enriched, whereas inflammatory factors (CCL21, IL-1a, WISP-1) declined. MMP-9, Galectin-1, and GDF-15 increased exclusively in the CD8-depleted group. **Conclusions:** The hADM protein content transitions from pro-inflammatory to pro-regenerative within one week after surgery. CD8^+^ T cell depletion accelerates this shift, highlighting hADM as a dynamic scaffold that contributes to the immune–regenerative crosstalk in bone healing.

## 1. Introduction

Large bone defects remain a significant challenge in trauma and orthopedic surgery. One treatment option is the induced membrane technique, a two-stage surgical procedure. In the first stage, filling the bone defect with polymethylmethacrylate (PMMA) induces a foreign body membrane with pro-regenerative properties. The second stage, performed after a variable interval of 6 weeks to 1 year, involves opening the induced membrane around the PMMA spacer, removing the spacer, and filling the defect with autologous bone material, followed by closure of the membrane around the defect [1]. This procedure could potentially be shortened to a single-stage process by using a substitute membrane in place of the induced membrane.

In previous projects, human acellular dermis (hADM) was identified as a potentially suitable replacement. Animal studies using a 5 mm femoral defect model in rats demonstrated equivalence to the induced membrane technique. Additionally, clinical cases have shown that this method can support healing of large bone defects [2,3]. However, hADM is initially acellular and primarily functions as a physical barrier in the early phases.

To overcome this limitation, hADM was preoperatively seeded with bone marrow mono-nuclear cells (BMC), which had previously been shown to significantly enhance bone defect healing when combined with a mineral scaffold and introduced into rat femoral defects [4]. Further studies applying the same animal model revealed a clear dose dependent effect of BMC for bone defect healing [5].

Unexpectedly, hADM seeded with BMC resulted in a significant impairment of bone defect healing, when wrapped around the bone defect [6]. Subsequent research attributed this negative effect to the population of CD8^+^ T cells within the BMC.

When hADM was seeded with BMC depleted of CD8^+^ T cells, significant improvements were observed in all measured parameters of bone healing compared to hADM seeded with complete BMC. Furthermore, immunohistological analyses revealed notable differences in the immunological environment in the defect region after eight weeks of healing between the two groups. Depletion of CD8^+^ T cells from BMC led to a marked reduction in CD8^+^ T cells throughout the defect area and decreased interferon (IFN)-γ expression within the defect [7]. CD8^+^ T cells and their secreted mediators, such as IFN-γ and tumor necrosis factor alpha (TNF-α), are known to negatively influence regenerative processes [8]. In that previous study, bone defect healing was evaluated as the endpoint. However, the early-phase events, which may be critical for determining the outcome of healing, remain largely unclear.

The aim of this study was to investigate how the protein composition of the human acellular dermal matrix (hADM) is modulated by colonization with bone marrow cells (BMC), either in the presence or absence of CD8^+^ T cells, during the early phase of bone healing (days three and seven in vivo). The protein content of the hADM is presumed to derive from multiple sources, including proteins released by the pre-seeded BMC, immigrating host cells, the surrounding tissue, blood infiltration, and the secretory activity of the fracture hematoma. Furthermore, we examined whether these protein alterations are associated with changes in the in vitro differentiation of macrophages and mesenchymal stem cells, two key regulators of bone regeneration.

## 2. Materials and Methods

### 2.1. Animal Care and Ethics

Eighteen 8 to 10 weeks old male Sprague-Dawley recipient rats (median weight: 340 g, interquartile range: 315–340 g; Janvier Labs, Saint Berthevin, France) were used in this study. Female rats were excluded from this study to avoid potential hormonal variations that could influence the interpretation of the results [9]. The rats were housed in cages of 3 to 4 rats each with free access to food and water in a light-controlled room (14 h light and 10 h dark per day) with a temperature range of 15 °C to 21 °C and air circulation. The animals were monitored daily after surgery and weekly thereafter. The animal experiments were registered and approved by the Animal Welfare and Monitoring Committee of the Regional Council ‘Regierungspräsidium’, Darmstadt, Germany (Approval Project No.FK/2020).

### 2.2. Study Design and Groups

The experiments were based on a 5 mm segmental femoral defect in Sprague-Dawley rats, which was filled with syngeneic cancellous bone graft from syngenic donor rats in all test subjects. For the harvest of cancellous bone, donor animals were euthanized under general anesthesia (3.5–4% isoflurane in air, followed by CO_2_). Tibiae and femora were excised, cleared of soft tissue, fragmented with bone forceps (fragment size 1–2 mm), and stored in PBS until implantation (within 1 h). The 5 mm defect was filled with approximately 100 µL bone fragments. The cancellous bone-filled defect was wrapped with a human acellular dermal matrix (hADM), which was differently pretreated depending on the group assignment.

In group 1 (G1, control), an unloaded hADM was used. In group 2 (G2), the hADM was seeded with bone marrow mononuclear cells (BMC), and in group 3 (G3), the hADM was seeded with the same number of CD8^+^ cell-depleted BMC. BMC were isolated from the bone marrow of long bones from syngeneic donor rats, and CD8^+^ cells in G3 were depleted using magnetic separation as described in following paragraphs. The number of animals per group and time point was *n* = 3, thus a total of 18 rats was analyzed.

**Figure 1:**
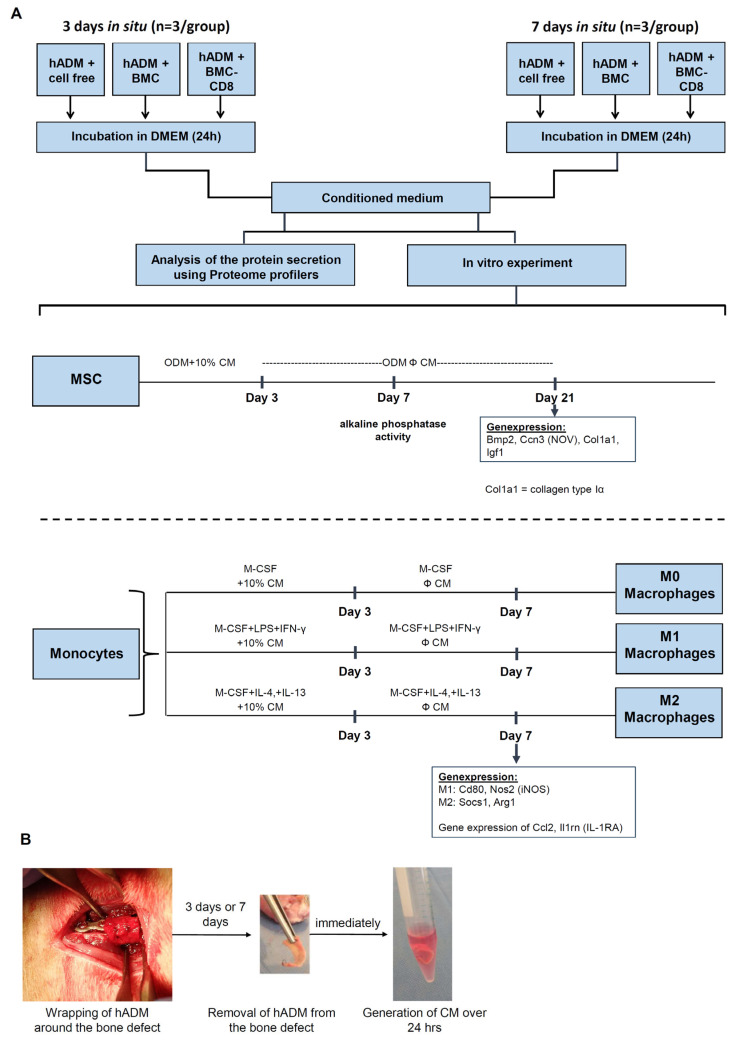
**Experimental design and workflow.** (**A**) Schematic overview of the study. Human acellular dermal matrices (hADM), either unseeded or pre-seeded with differentially pretreated BMC, were implanted around a femoral defect for 3 or 7 days. Explanted hADM were subsequently used to generate conditioned media (CM). Secreted proteins were analyzed semi-quantitatively using a proteome profiler. The biological activity of CM was then assessed in vitro with respect to osteogenic differentiation of mesenchymal stem cells (MSC) and polarization of macrophage subtypes. (**B**) Illustration of hADM wrapped around the bone defect, the subsequent explantation, and the generation of CM.

After 3 or 7 days, the animals were euthanized, and the hADM surrounding the defect was carefully explanted. The explanted hADM was incubated in serum-free medium for 24 h to produce conditioned medium (CM). The CM was analyzed using a proteome profiler, and its effects on the differentiation of rat mesenchymal stem cells (MSC) and macrophages were assessed (Figure 1). These time points are potentially biologically meaningful to assess early immune dynamics that influence later bone healing outcomes. Within the first 72 h post-injury, innate inflammation peaks and adaptive immune cells, including T cells, begin to infiltrate the fracture hematoma, with T cells becoming activated and modulating local cytokine milieus by day 3. Adaptive subsets such as Th17 and other effector T cells continue to shape the microenvironment through day 7, a period when crosstalk with MSCs and macrophages begins to direct the transition from inflammation to repair. Dysregulated CD8^+^ T-cell activity during this window has been linked to impaired regenerative responses [10].

### 2.3. Isolation of BMC and Colonization of hADM with BMC or BMC-Depleted CD8^+^ T Cells

BMC were isolated from the bone marrow of long tubular bones of syngeneic donor rats. BMC were used for the colonization of hADM, but also for the isolation of MSC and bone marrow-derived macrophages. In brief, donor rats (*n* = 12) were euthanized by an overdose of pentobarbital and the femora and tibiae were harvested under sterile conditions. The bones were transferred to phosphate-buffered saline solution without Mg^2+^/Ca^2+^ (PBS^-/-^, Gibco, Paisley, UK) containing 5% penicillin/streptomycin (Sigma-Aldrich, St. Louis, MO, USA). Next, the condyles were cut, the bone marrow was flushed out of the bone using a syringe filled with PBS^-/-^ and 1% penicillin/streptomycin, followed by suspension through repeated pipetting. Finally, the bone marrow cell suspension was filtered through a 70 µm cell strainer (BD-Biosciences, Heidelberg, Germany) and washed twice with PBS^-/-^ (centrifugation conditions: 300× *g*, 5 min at room temperature). Subsequently, BMCs were isolated by density gradient centrifugation (Biocoll^®^ separation solution density 1.077 g/cm^3^, Biochrom, Berlin, Germany (centrifugation: 30 min, 400× *g*, 20 °C without brake). These BMC served as cellular source for all subsequent experiments.

### 2.4. Depletion of CD8^+^ T-Cells from BMC

CD8^+^ cells from the BMC fraction (10^7^ cells) were depleted by positive magnetic selection applying anti-rat CD8α MicroBeads (130-090-318, Miltenyi Biotec, Bergisch Gladbach, Germany) and LD columns (Miltenyi Biotec) thereby following the instructions of the manufacturer and described in [7]. The effectiveness of the depletion was confirmed by FACS analysis. The CD8^+^ cells were reduced by a factor of 35, as documented in our prior preliminary study [7].

### 2.5. Seeding of hADM with BMC

The hADM (Epiflex^®^) is derived from the skin of serologically tested donors and manufactured using validated processes, including decellularization, sterilization, and tissue preservation [11]. It is approved as a medicinal product in accordance with §21 of the German Medicinal Products Act (approval number: 3003749.00.00).

HADM was colonized with either complete BMC or CD8^+^ cell-depleted BMC. The hADM (0.8 mm thickness; German Institute for Cell and Tissue Replacement, DIZG, Berlin, Germany, https://dizg.de/en/allograft/epiflex-0-3-mm-3-mm/, accessed on 21 January 2026) was cut into 1 × 2 cm pieces using a template and pre-wetted for 10 min in PBS containing Mg^2+^/Ca^2+^ at room temperature under sterile conditions. Each hADM piece was loaded with 1.125 × 10^6^ cells for 10 min at 37 °C, then centrifuged five times at 300× *g* for 1 min each, inverted, and reloaded with 1.125 × 10^6^ cells. The prepared hADM specimen, containing each a total of 2.25 × 10^6^ cells, were preserved in PBS with Mg^2+^/Ca^2+^ and implanted immediately after cell loading [7].

### 2.6. Surgical Procedure

The surgical procedure was performed as previously described [2,12,13]. For anesthesia, the animals received 2 mL of Ketavet (70 mg/kg) and Rompun (10 mg/kg) intraperitoneally. After shaving and aseptic cleaning of the right hind limb, a longitudinal incision was made over the femur. The muscles were separated, and a locking compression plate (Miniplate “Lockingplate LCP Compact Hand 1.5 straight”, DePuySynthes, Dubendorf, Switzerland) was fixed to the anterior femoral shaft. Four screws were used to secure the plate, and a 5 mm defect was created in the femoral shaft using a Gigli saw. According to the group set up, differentially pretreated hADM was wrapped around the defect and filled with 100 µL of vital bone chips obtained directly before surgery from syngenic donor rats. The wound was closed with Vicryl and Prolene sutures. Postoperative analgesia (Carprofen, 2.6 mg/kg s.c.) was given daily over at least 6 days postoperatively (D7 group).

### 2.7. Production of Conditioned Media (CM)

The animals were euthanized after three days or seven days, and femurs were harvested for isolation of the previously implanted hADM. The hADM, with and without cells, was dissected from the right femur, placed in a tube containing 2 mL of DMEM, and incubated for 24 h at 37 °C in an incubator. The hADM was then removed from the tube, and the medium was centrifuged at 2300× *g* for 15 min at 4 °C to remove cells and debris. The resulting CM (listed in Table 1) was stored at −80 °C in 1 mL aliquots for proteome profile analysis and cell differentiation experiments.

### 2.8. Analysis of Protein Release from CM Using Proteome Profilers

The Proteome Profiler Rat XL Cytokine Array (R&D Systems, Minneapolis, MN, USA) was used to detect 79 mediators simultaneously and investigate the inflammatory and angiogenic potential of the hADM in a time-dependent manner, depending on the cell load. The procedure was performed according to the manufacturer’s instructions (see details in [14]. In brief, each membrane was treated with blocking buffer for 1 h, followed by overnight incubation with diluted CM samples (600 µL CM and 900 µL array buffer) at 2–8 °C on a rocking platform shaker. The next day, the membranes were washed three times with wash buffer (10 min per wash), then incubated with a cocktail of biotinylated detection antibodies (30 µL antibody and 1.5 mL buffer) for one hour at room temperature. After removing unbound proteins by washing, 2 mL of 1× streptavidin HRP was added to the membrane and incubated for 30 min at room temperature. Following another wash step, 1 mL of the chemiluminescent reagent mixture was pipetted evenly onto the membrane corners and incubated for 1 min. Finally, the membranes were exposed for 5 min using a Fusion Fx7 gel scanner equipped with a high dynamic range digital camera (Vilber Lourmat, Eberhardzell, Germany). The density of the spots from the samples was evaluated in relation to the reference spots using ImageJ (https://imagej.net/, accessed on 21 January 2026).

### 2.9. Cultivation, Osteogenic Differentiation, and CM Treatment of MSC

Mesenchymal stem cells (MSCs) and macrophages were isolated from BMCs obtained from the femoral and tibial bones of donor rats. The isolation of both cell types was based on their different adhesion behavior. In brief, BMC obtained from femora and tibiae of one donor rat were suspended in each 12 mL of medium (Dulbecco’s Modified Eagle Medium, DMEM + GlutaMAX + 1 g/L D-glucose, Gibco, Paisley, UK). After 24 h in a 75 cm^2^ cell culture flask under humid conditions at 37 °C and 5% CO_2_, the MSC adhered to the surface of the culture vessel and grew slowly, while the erythrocytes, lymphocytes, and monocytes remained suspended in the culture medium.

After removal of the non-adherent fraction following the initial 24 h culture of BMC, the adherent cell fraction enriched in MSC was further expanded in MSC growth medium (MSC-GM, DMEM + GlutaMAX + 1 g/L D-glucose) supplemented with 10% heat-inactivated fetal bovine serum (FBS, Gibco) and 100 U/mL penicillin/100 µg/mL streptomycin (Sigma-Aldrich).

MSC-GM was replaced twice weekly, and cells were passaged upon reaching ~80% confluence. For passaging, MSC monolayers were washed once with PBS, detached using Accutase (Sigma-Aldrich) at 37 °C for 5–7 min, and the reaction was stopped by adding MSC-GM. Cells were then centrifuged (300× *g*, 5 min, room temperature), recovered, and expanded to passage 2.

The osteogenic differentiation potential of MSC was verified as previously described [15,16,17]. For experiments, passage-2 MSC were seeded in 24-well plates (Sarstedt, Nümbrecht, Germany) at a density of 2 × 10^4^ cells/mL per well in osteogenic differentiation medium (ODM) containing 10% (*v*/*v*) conditioned medium (CM) and cultured for 3 days. After medium exchange (day 4), MSC were further maintained for either 7 or 21 days in ODM (MSC-GM supplemented with 10^−6^ M dexamethasone and 10 mM β-glycerophosphate (both Sigma-Aldrich), and 0.05 mM ascorbic acid (Stemcell Systems, Cologne, Germany)) or in MSC-GM as a negative control (Figure 1). At each time point, cells were harvested for analysis of osteogenic differentiation. Osteogenic differentiation was assessed based on measurement of alkaline phosphatase activity, and the gene expression of bone morphogenetic protein (BMP)-2, collagen type Iα (Col1a1, see Section 2.11).

The activity of alkaline phosphatase in the samples was determined after 7 days of culture (Figure 1) using the SensoLyte^®^ pNPP Alkaline Phosphatase Assay Kit (AnaSpec, Inc., Fremont, CA, USA) as a colorimetric assay, following the manufacturer’s protocol. In brief, 200 µL of assay buffer with 0.2% Triton X-100 were added to the cells, which were then detached from the surface by scraping. The samples were incubated on ice for 30 min with constant shaking, followed by centrifugation at 10,000× *g* and 4 °C for 15 min. The supernatant from each sample and standard was pipetted in duplicate (50 µL/well) into a 96-well plate. Then, 50 µL of substrate solution (pNPP) was added, and the samples were incubated for 45 min at room temperature. Absorbance was measured at 405 nm. Gene expression of osteogenic marker was assessed as described in the Section 2.11.

### 2.10. Isolation, Polarization, and Treatment of Bone Marrow Derived Macrophages with CM

For isolation of monocytes the non-adherent cell fraction was harvested by centrifugation (300× *g*/5 min/room temperature) after the initial 24 h culture of BMC. The medium was removed and stored. The cell pellet was washed, counted and resuspended in the previously collected medium and placed in a 24-well plate with a cell density of 7 × 10^6^ cells/mL per well [18]. The bone marrow derived macrophages (BMM) required a further 48 h to adhere to the bottom of the culture flasks. After this period, the medium was carefully removed and replaced with fresh RPMI 1640 medium (+L Glutamin, Gibco) containing 10% FBS, 1% P/S and 50 ng/mL macrophage colony-stimulating factor (M-CSF, PeproTech^®^, Rocky Hill, NJ, USA) to differentiate bone marrow progenitor cells into M0 macrophages (M0 medium). Polarization to M1 macrophages was achieved using M0 medium with additional stimulation by 20 ng/mL IFN-γ (PeproTech^®^) and 100 ng/mL lipopolysaccharide (LPS, Sigma-Aldrich), while M2 macrophages were induced using M0 medium with additional stimulation by each 20 ng/mL IL-4/IL-13 (PeproTech^®^) [19]. Each culture of the macrophage subgroups (M0, M1, M2) also received 10% CM and was cultured for three days. After this period, the medium was replaced with fresh medium (without CM), the cells were cultivated for a further four days and lysed for gene expression analysis (see below). Monocytes cultured with their respective differentiation media but without addition of CM served as control.

### 2.11. Gene Expression Analyses

Total RNA was isolated using the RNeasy Micro Kit system (Qiagen, Hilden, Germany) thereby following the instructions of the manufacturer. In brief, MSCs (collected on day 21 after start of differentiation) and macrophages (collected on day 7 after start of differentiation) were lysed with 350 µL of lysis buffer (Qiagen, Hilden, Germany) containing guanidinium thiocyanate and 1% β-mercaptoethanol, then homogenized using an insulin syringe and stored at −80 °C for future use. RNA concentration and quality were determined employing a Nanodrop NanoVue Plus (Biochrom, Holliston, MA, USA). RNA was diluted with RNase-free water to 100 ng/μL, and cDNA synthesis was performed using the AffinityScript system (Agilent Technologies, Santa Clara, CA, USA) according to the manufacturer’s protocols. Gene expression levels of characteristic markers (Immune cell markers and immune regulators for macrophages, bone-specific markers for MSCs) were semi-quantitatively assessed using a SYBR-green based approach applying the CFX96 Touch Real-Time PCR Detection System (Bio-Rad, Hercules, CA, USA). Data were normalized to the expression of the housekeeping GAPDH (2^−ΔCt^-method). Specific primers were obtained from Qiagen (Table 2).

### 2.12. Statistical Analysis

Statistical analyses were performed using Bias software (version 11.10, Epsilon, Weinheim, Germany), and data were expressed as medians with minimum and maximum in figure and median with 25% and 75% quartiles in tables. Differences between groups in protein levels and gene expression were assessed using the nonparametric unpaired Wilcoxon–Mann–Whitney U test, which does not assume any specific data distribution. Due to the small sample size per group (*n* = 3) no correction for multiple testing (e.g., Bonferroni adjustment) was applied, as this would markedly increase the risk of type II errors in this small-scale experimental setting. Instead, the reported *p*-values of pairwise comparisons are considered exploratory and should be interpreted with caution, acknowledging the increased risk of type I errors. This approach follows the rationale discussed by Bender & Lange (2001) and Rothman (1990), emphasizing the value of avoiding over-conservative adjustments in exploratory studies [20,21]. To determine the direction of the observations, *p*-values less than 0.05 were considered significant, and *p*-values less than 0.1 were regarded as indicative of a trend. These thresholds were mainly used to describe the data. Heatmap was generated with FunRich 3.1.3 [22]. Pie charts were generated with MS-Excel, diagrams for protein and gene expression with Graphpad Prism 10.0.

## 3. Results

### 3.1. Changes in Protein Secretion Patterns over Time

hADMs were used either uncolonized, colonized with BMC, or colonized with BMC-CD8, and subsequently wrapped around bone defects in vivo for 3 or 7 days. Explanted hADMs were incubated in serum-free medium for 24 h to generate CM, which was analyzed using a proteome profiler detecting 79 proteins and further tested for its effects on MSC osteogenic differentiation and macrophage polarization.

**Figure 2:**
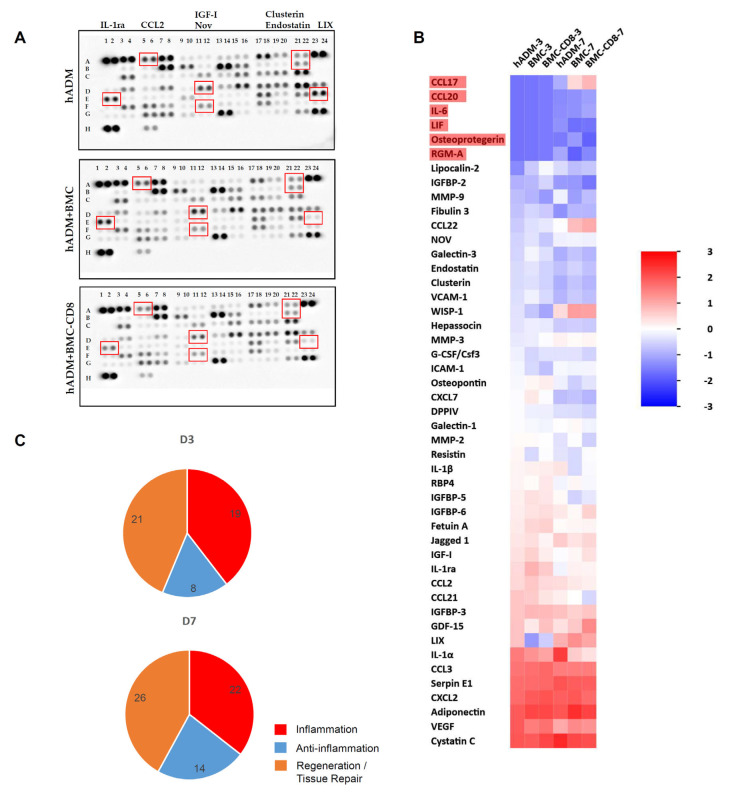
**Semiquantitative evaluation of protein expression using proteome profiler arrays.** Representative protein profiler arrays of defects treated with hADM, hADM + BMC, or hADM + BMC-CD8 after three days are shown in (**A**). The differentially expressed proteins are marked by a red square. Protein expression was analyzed denitometrically and spot density was normalized to control spots located in the upper left, upper right, and lower left corners. Heatmap generated on basis of protein expression is shown in (**B**). Heatmap contains data from differentially pretreated hADM recovered on D3 and 7. Proteins newly secreted on D7 are marked in red. Color code indicates fold change. The pie charts (**C**) show the number of secreted proteins from membranes that covered the bone defect for 3 (upper pie chart) and 7 days (lower pie chart). The categories inflammation (red), anti-inflammation (blue) and regeneration (orange) are shown. Bi- or multifunctional proteins were assigned to the respective categories, so that the sum of the fractions is higher than the total number of proteins detected. There was a general increase in the number of expressed proteins from D3 to D7. Detected proteins and their main biological functions is listed in Supplementary Table S1.

Overall, the aim was to identify proteins whose secretion was influenced by treatment type and incubation time. After three days in vivo, 41 of 79 proteins were detectable in CM, while after seven days, 47 proteins were detected, regardless of the cellular loading of the hADMs. The secretory profile changed over time: RGM-A, osteoprotegerin, LIF, IL-6, CCL20, and CCL17 were exclusively secreted by hADMs after seven days. These changes suggest a shift toward tissue regeneration and anti-inflammatory activity at day seven (Figure 2). Functions and cellular sources of the secreted proteins are summarized in Supplementary Table S1.

### 3.2. Effect of BMC Loading of hADM on Protein Content After Three and Seven Days In Vivo

**Figure 3:**
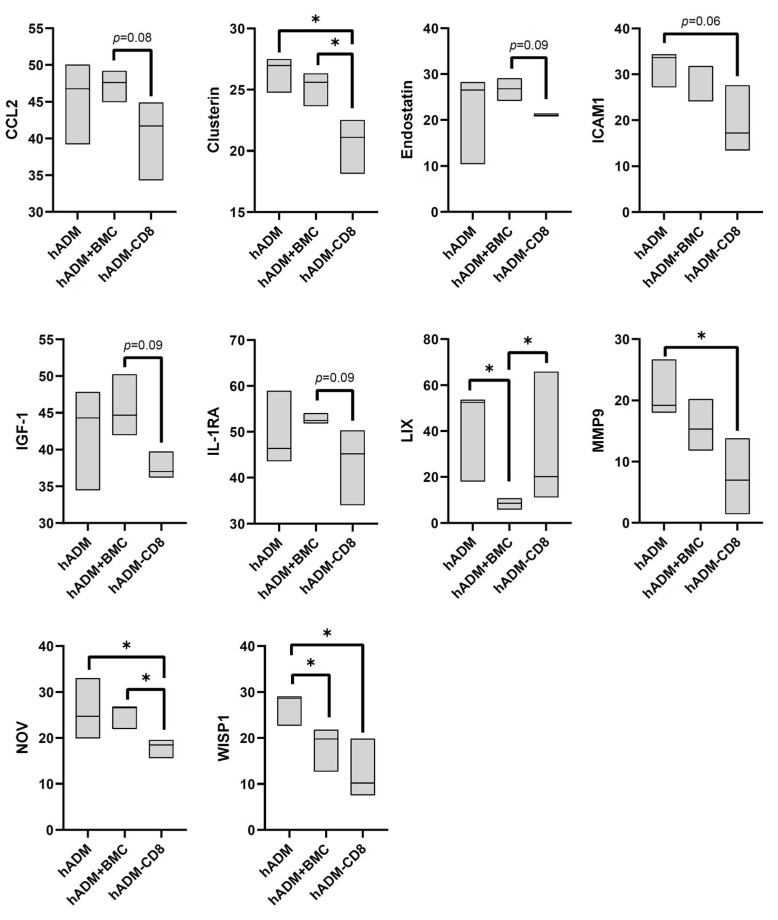
**Protein profiler analysis of conditioned media from defects treated with hADM, hADM + BMC, or hADM + BMC-CD8 after three days.** Protein levels are normalized to the intensity of the control spots (=100%). Only proteins with significant differences (*p* < 0.05) or trends (*p* < 0.1) are shown. Data are presented as boxplots (median, minimum, maximum; *n* = 3 per group). * indicates explorative significance (*p* < 0.05) versus the respective comparison group.

On day three after implantation, hADM alone showed increased release of LIX and WISP compared with hADM colonized with BMC. Depletion of CD8⁺ cells from BMC lead to decreased secretion of clusterin, ICAM-1, MMP-9, NOV, WISP-1 compared to hADM and to reduced expression of CCL2, clusterin, endostatin (Col18α1), IGF-1, IL-1RA and NOV compared to hADM + BMC while LIX secretion was increased relative to hADM + BMC (Figure 3).

At day seven post implantation, hADM alone showed increased secretion of IL-1alpha and RGM-A compared with hADM colonized with BMC, and elevated CCL21 and IL-1alpha secretion relative to hADM + BMC-CD8.

**Figure 4:**
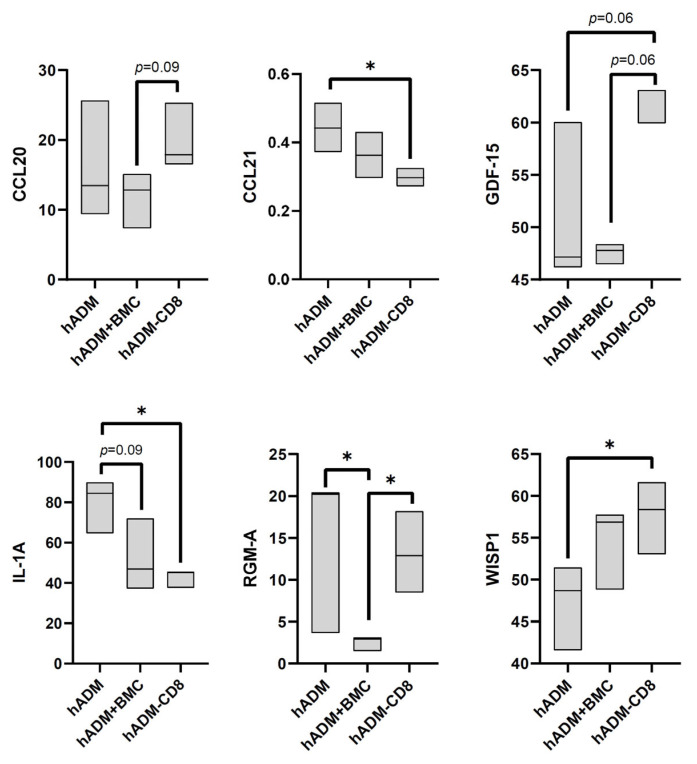
**Protein profiler analysis of conditioned media from defects treated with hADM, hADM + BMC, or hADM + BMC-CD8 after 7 days**. Protein levels are normalized to the intensity of the control spots (=100%). Only proteins with significant differences (*p* < 0.05) or trends (*p* < 0.1) are shown and presented in alphabetical order. Data are presented as boxplots (median, minimum, maximum; *n* = 3 per group). * indicates explorative significance (*p* < 0.05) versus the respective comparison groups.

Secreted CCL20, GDF-15 and RGM-A levels were higher in hADM + BMC-CD8 compared with hADM + BMC (Figure 4).

### 3.3. Temporal Comparison of Secreted Proteins Between 3 and 7 Days In Vivo

**Figure 5:**
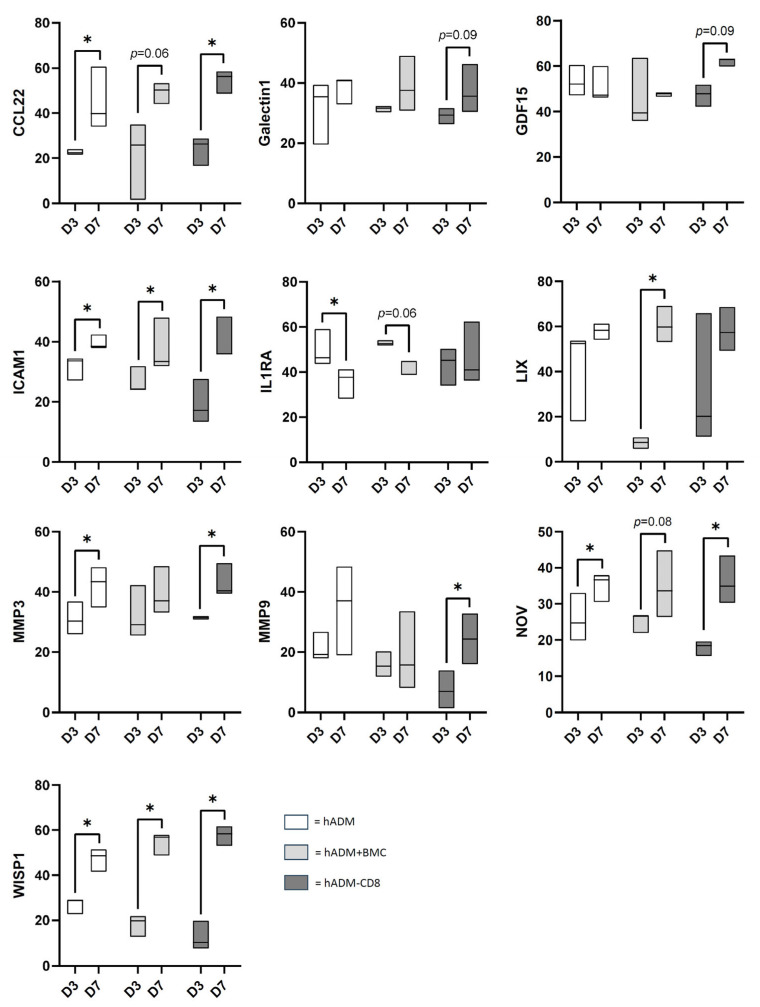
**Direct comparison of protein profiler data obtained after 3 and 7 days in defects treated with hADM (white), hADM + BMC (light gray), or hADM + BMC-CD8 (dark gray).** Values are normalized to the density of the control spots (=100%). Only proteins with significant differences (*p* < 0.05) or trends (*p* < 0.1) are shown and presented in alphabetical order. Data are presented as boxplots (median, minimum, maximum; *n* = 3 per group). * indicates explorative significance (*p* < 0.05) versus the respective comparison group.

Compared to the three-day treatment, levels of CCL22, ICAM-1, NOV and WISP-1 were elevated after seven days across all treatment groups, with the strongest increase observed in the hADM + BMC-CD8 group. In contrast, expression of Galectin-1, GDF-15, MMP-3 and MMP-9 was increased after seven days only in the hADM + BMC-CD8 group. For LIX, an upregulation was detected exclusively in the hADM + BMC group, while MMP-9 exhibited a slight increase in the hADM group. Notably, IL-1RA levels declined after seven days compared to three days in both the hADM and hADM + BMC groups (Figure 5).

### 3.4. Influence of CM Generated with Differentially Treated hADM on Osteogenic MSC Differentiation

Primary rat mesenchymal stem cells (MSCs) were cultured for 21 days in osteogenic differentiation medium supplemented with 10% conditioned medium (CM) derived from the different treatment groups. Subsequently, expression levels of the osteogenic genes *Bmp2*, *Col1a*, *Igf1*, and *Ccn3* (Nov) were analyzed.

Overall, CM obtained from human acellular dermal matrices (hADM) frequently modulated MSC gene expression compared to control cultures without CM. These effects were more pronounced when CM was derived from hADM after three days of treatment than after seven days.

**Figure 6:**
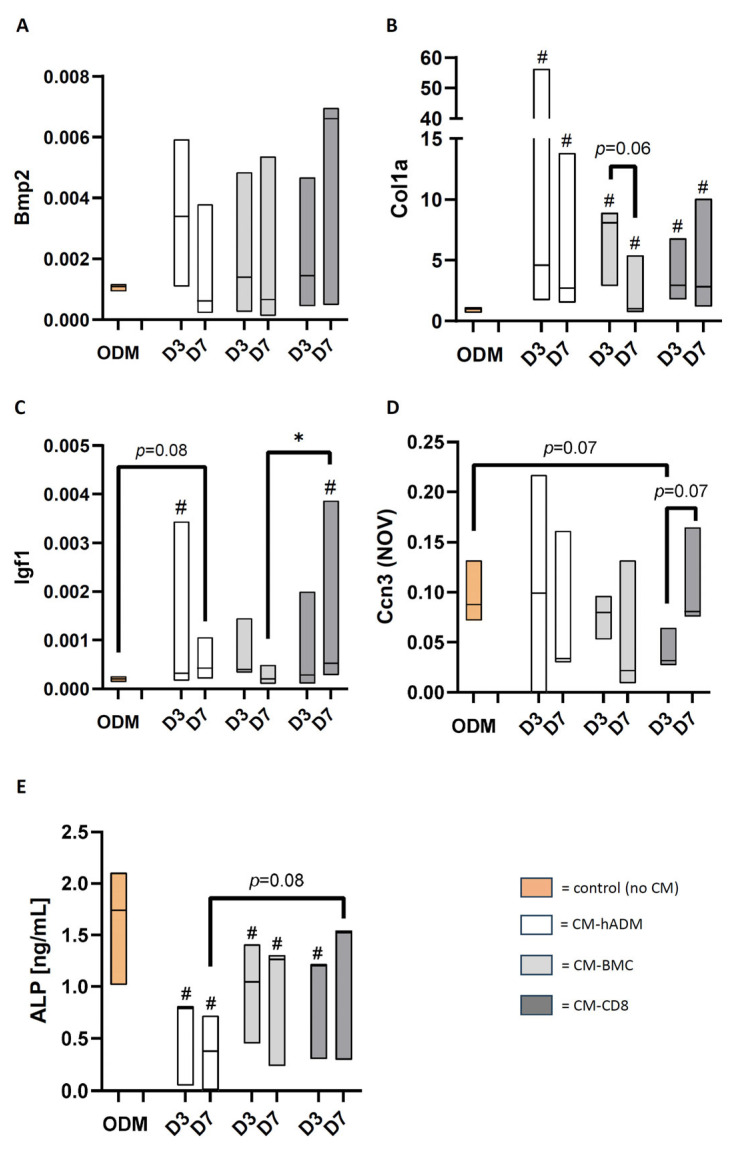
**Osteogenic gene expression (A–D) and ALP protein expression (E) in MSC after cultivation in osteogenic differentiation medium (ODM) supplemented with CM.** CM was generated from hADM (white), hADM seeded with BMC (light gray), or hADM seeded with CD8-depleted BMC (BMC-CD8, dark gray) wrapped around the bone defect for 3 or 7 days, then explanted and incubated in medium for 24 h. MSC were cultured for 3 weeks in ODM, with 10% CM added during the first 3 days, followed by analysis of gene expression relative to the Gapdh housekeeping gene (**A**–**D**) or ALP measurement (**E**). Control MSC (salmon) were cultured in expansion medium (M, only in **E**) or in ODM, both without CM. # = *p* < 0.05 vs. ODM; * = *p* < 0.05 vs. indicated group. All significances are exploratory.

Notably, *Col1a* gene expression was consistently upregulated by CM from both the three-day and seven-day treatment groups, irrespective of cellular loading, with the exception of CM-BMC-7. Among the other genes, *Igf1* expression was significantly increased in response to CM-BMC-3, CM-hADM-3, and CM-CD8-7, compared to the control. In contrast, Nov was the only gene significantly downregulated by CM treatment, with CM-CD8-3 leading to a suppression relative to the control (Figure 6).

Alkaline phosphatase activity of osteogenically induced MSCs cultured with different conditioned media (CM) was assessed after seven days in OD medium. Alkaline phosphatase levels were significantly reduced in all CM-treated cultures compared to the control cultured in OD medium without CM, with the exception of CM-CD8-7. Notably, ALP activity was tendentially higher in MSCs stimulated with CM-CD8-7 compared to CM-hADM-7 (Figure 6).

### 3.5. Influence of CM Generated with Differentially Treated hADM on Macrophage Polarization

Macrophages were exposed to conditioned media (10%; Figure 1, Table 1) during a three-day stimulation phase, followed by a seven-day differentiation period without CM towards M0, M1 or M2 subtype. Gene expression of M1 (*Cd80*, *Nos2*) and M2 (*Arg1*, *Socs1*) lineage markers was subsequently assessed by RT-qPCR.

### 3.6. Expression of M1 and M2 Marker Genes in M0, M1 and M2 Differentiated Macrophages

Conditioned medium (CM) had different effects on the expression of M1 and M2 marker genes in M0-differentiated macrophages, depending on cell loading and implantation period. For example, CM-hADM-7 significantly induced the expression of *Nos2* and *Arg1* compared with the control. In contrast, CM-CD8 showed only a minor stimulatory effect on the investigated genes.

During M1 differentiation, incubation with the various CMs did not result in clear changes in *Cd80* or *Nos2* gene expression compared with macrophages cultured without CM.

**Figure 7:**
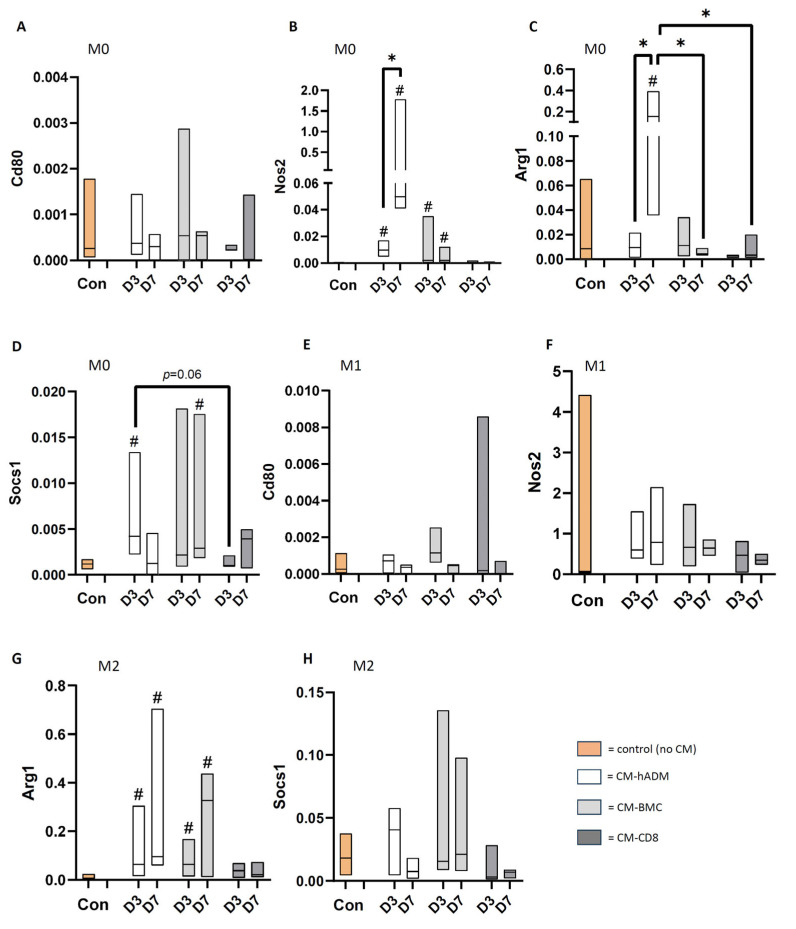
**Influence of CM on expression of M1 (*Cd80, Nos2*) and M2 (*Arg1, Socs1*) marker genes in macrophages differentiated into M0 (A–D), M1 (E,F), or M2 (G,H).** CM was generated from hADM (white), hADM seeded with BMC (light gray), or hADM seeded with CD8-depleted BMC (BMC-CD8, dark gray) wrapped around the bone defect for 3 or 7 days, then explanted and incubated in medium for 24 h. Primary rat monocytes from bone marrow aspirates were cultured for 7 days, with 10% CM added during the first 3 days. Monocytes cultured in differentiation media without CM served as controls (salmon). # = *p* < 0.05 vs. control; * = *p* < 0.05 vs. indicated group. All significances are exploratory.

In M2-differentiated macrophages, incubation with CM-hADM-3 and -7 as well as CM-BMC-3 and -7 led to a significant induction of Arg1 expression. This effect was markedly reduced for CM-CD8-3 and -7. *SOCS1* expression was not significantly influenced by any of the CMs; however, the lowest values were also observed after incubation with CM-CD8-3 and -7 (Figure 7).

### 3.7. CCL2 and IL-1RA Gene Expression in M1- and M2-Differentiated Macrophages

**Figure 8:**
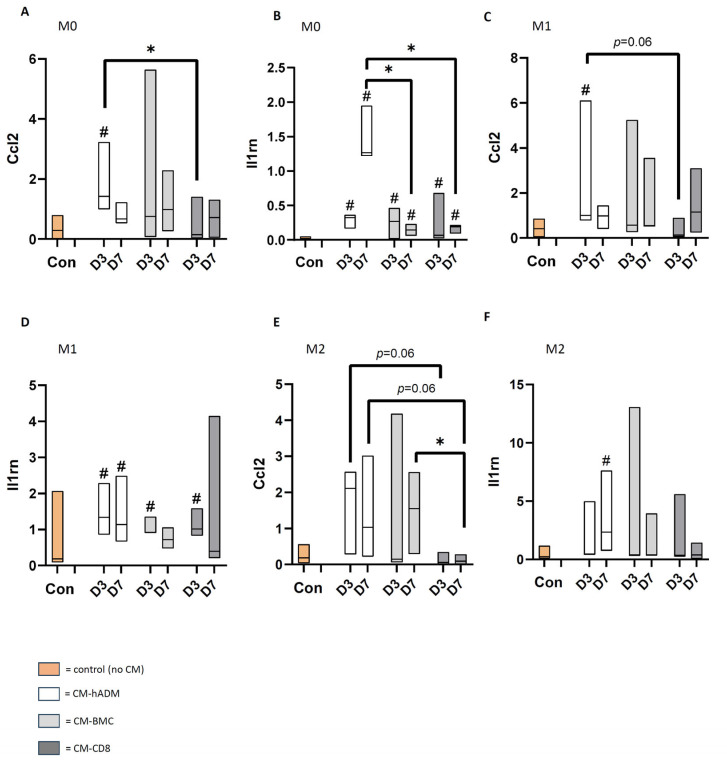
**Influence of CM on expression of *CCL2* and *IL-1RA* genes in macrophages differentiated into M0 (A,B), M1 (C,D), or M2 (E,F).** CM was generated from hADM (white), hADM seeded with BMC (light gray), or hADM seeded with CD8-depleted BMC (BMC-CD8, dark gray) wrapped around the bone defect for 3 or 7 days, then explanted and incubated in medium for 24 h. Primary rat monocytes from bone marrow aspirates were cultured for 7 days, with 10% CM added during the first 3 days. Monocytes cultured in differentiation media without CM served as controls (salmon). # = *p* < 0.05 vs. control; * = *p* < 0.05 vs. indicated group. All significances are exploratory.

To investigate whether the cytokine released by the differentially pretreated hADMs influence the pro- and anti-inflammatory reaction of macrophage subtypes, gene expression of *CCL2* (pro-inflammation, cell recruitment) and IL-1RA (neutralization of IL-1) was assessed in M0-, M1-, and M2-differentiated macrophages in vitro. Interestingly, incubation with CM-hADM-3 lead to a significant induction of *CCL2* gene expression in M0- and M1-differentiated macrophages and an increase (not significant) in M2-differentiated macrophages. *CCL2* gene expression was downregulated by CM-CD8-3 in M0, M1, and M2 macrophages compared to CM-hADM-3. For *IL-1RA*, gene expression in M0 and M1 macrophages was increased in all CM-treated groups compared to control without CM. In M2 macrophages, *IL-1RA* gene expression was elevated only after stimulation with CM-hADM-7 compared to control (Figure 8).

## 4. Discussion

This study was conducted on the background that hADM loaded with CD8-depleted BMC led to a significant improvement of the bone healing outcome with simultaneously lower inflammatory activity in the bone defect area compared to hADM with complete BMC or unloaded hADM [7]. The application of hADM seeded with CD8-depleted BMCs as a wrapping for large bone defects is conceptually derived from the induced membrane technique (Masquelet technique). In the classical approach, a foreign body membrane is generated in a first surgical stage, creating a biologically active chamber that supports subsequent bone regeneration [23,24,25].The use of a preformed artificial membrane—potentially further functionalized with cells possessing regenerative capacity—may allow this membrane induction phase to be bypassed. Such an approach has the potential to reduce the number of surgical procedures and substantially shorten the overall treatment period. Both strategies were directly compared in a rat femoral defect model. The use of hADM as a defect-wrapping membrane resulted in bone defect healing comparable to that achieved with the conventional induced membrane technique, while requiring only a single surgical intervention and shortening the treatment duration by approximately 3–4 weeks due to the omission of the foreign body membrane induction phase [2]. In the same animal model, the additional seeding of hADM with CD8-depleted BMCs led to a further improvement in multiple bone healing parameters compared with unseeded hADM [7]. These findings suggest that the use of a biologically functionalized artificial membrane may not only replicate but potentially enhance the regenerative microenvironment typically established by the induced membrane in the Masquelet technique.

However, the healing of large bone defects requires a fine-tuned regulation of regenerative, inflammatory, and anti-inflammatory processes. There is increasing evidence that terminally differentiated, CD8-positive, cytotoxic T cells in particular exert a negative influence on regenerative processes and, in particular, on bone healing [26]. Against this background, the present study investigated the secretome of hADM—either unloaded, loaded with BMC, or with CD8-depleted BMC—after implantation into cancellous bone-filled defects for three or seven days. The conditioned medium derived from these hADM grafts was further used to study effects on MSC osteogenic differentiation and macrophage polarization.

### 4.1. BMC as Cellular Therapeutic

In this study, bone marrow cells (BMCs) were used as a cellular therapeutic in combination with the human acellular dermal matrix (hADM). BMCs were selected because they can be harvested with relative ease and have demonstrated regenerative potential in various tissues. A key advantage of BMCs is their logistical suitability for use in the acute treatment of large bone defects, as they can be applied without extensive ex vivo manipulation [27,28]. However, this benefit is accompanied by increased immunological activity at the transplantation site, which is attributable, among other factors, to the presence of cytotoxic T lymphocytes within the BMC population [7].

In contrast, mesenchymal stromal cells (MSCs) are currently not suitable for acute clinical application due to the time required for cell expansion in culture. Recent developments, however, suggest that this limitation may be overcome by the use of defined MSC pools. These MSC pools are non-immunogenic and are already being used therapeutically to control graft-versus-host disease. Thus, an off-the-shelf cellular source for MSC-based therapies could become available without the need for time-consuming expansion of autologous MSCs [29,30,31].

It has been shown that collagenous membranes can be efficiently colonized by MSCs [32]. When applied to the system described here, it is conceivable that the immunomodulatory properties of MSCs could attenuate the activity of cytotoxic T lymphocytes, thereby promoting a more favorable healing response. Conversely, there is evidence that MSCs may also impair the activity of pro-regenerative T helper cells, which could negatively affect tissue regeneration [33]. In addition to their immunomodulatory effects, MSCs may also be directly involved in the formation of new bone tissue. Several studies have demonstrated that collagen type I—the predominant collagen in the dermis—promotes the osteogenic differentiation of MSCs [34].

The temporal profile of protein factors released from an MSC-seeded collagenous membrane remains speculative. Given the immunosuppressive properties of MSCs, it is reasonable to assume a reduced presence of pro-inflammatory mediators. Furthermore, an increased release of proteins primarily associated with MSC activity and osteogenic differentiation can be anticipated, including bone morphogenetic proteins (BMPs), transforming growth factor-β (TGF-β), stromal cell-derived factor-1 (SDF-1), vascular endothelial growth factor (VEGF), as well as alkaline phosphatase (ALP), collagen type I alpha (COL1A), and osteopontin [35].

### 4.2. Sources of Proteins Released by the hADMs

An important point concerns the source of proteins in the hADMs. The experiments showed that the overall composition of proteins released by differently pretreated hADMs varied only slightly. Since the initially cell-free hADM also released proteins to a comparable extent, it can be assumed that a relevant proportion of the released proteins originates from the recipient animal. Possible sources include cells introduced by bleeding during and after surgery (as it can be seen on the left image in Figure 1B), active cell migration, uptake of secreted proteins from the defect site and surrounding tissue (“sponge function”). Due to its large internal surface area, hADM has a high capacity for cell loading, and its fibrous collagenous structure provides numerous binding sites. A rapid postoperative loading of hADM with cells and proteins could explain why even initially cell-free hADM enables a significant healing effect [2].

### 4.3. Dynamics of the Protein Release: Factors Secreted Exclusively by D7 Samples

Retention time in vivo had a marked influence on the hADM protein release. More proteins were detectable at day seven compared to day three, independent of cell loading. This indicates a temporal shift from inflammation toward regeneration and resolution of inflammation. Several mediators—RGM-A, osteoprotegerin, LIF, IL-6, CCL20, and CCL17—were exclusively detected after seven days. These proteins fulfill complementary roles in modulating inflammation, cell recruitment, angiogenesis, and bone remodeling.

Repulsive Guidance Molecule A (RGM-A) is a glycosylphosphatidylinositol-anchored co-receptor that modulates BMP signaling by binding BMP ligands and facilitating their interaction with BMP receptors, thereby enhancing SMAD-dependent transcriptional activity. RGMs are known to fine-tune BMP pathway responsiveness, which is critical for osteogenic commitment and early bone formation [36]. Although most research on RGM-A has focused on neural and inflammatory contexts, its involvement in BMP signaling suggests it could influence early skeletal repair by modulating local BMP-mediated osteoprogenitor differentiation and inflammation, key processes in the initial stages of fracture healing [37].

Furthermore, it mitigates inflammation and leukocytes (granulocytes, monocytes) as well as T- and B-lymphocytes. It also inhibits leukocyte migration by contact repulsion and chemorepulsion, depending on dosage, through its receptor neogenin [38].

Osteoprotegerin (OPG) is a decoy receptor for RANKL that is expressed by osteoblasts and stromal cells and acts to inhibit osteoclastogenesis, thereby limiting bone resorption through the RANK/RANKL/OPG pathway. During fracture healing, OPG expression increases rapidly, with an early peak within the first 24 h, suggesting a role in modulating the initial balance between resorption and formation and in protecting nascent callus from excessive osteoclast activity. The early OPG response likely contributes to establishing a favorable microenvironment for subsequent bone formation and remodeling [39,40].

Interleukin-6 (IL-6) is a key pro-inflammatory cytokine that is rapidly upregulated at fracture sites during the acute inflammatory phase. It contributes to the recruitment and activation of immune cells, supports angiogenesis via VEGF induction, and regulates early osteoblast and osteoclast differentiation essential for callus formation. Knockout studies demonstrate that IL-6 deficiency delays callus mineralization and maturation, highlighting its importance in early bone repair. IL-6 may also influence MSC osteogenic responses and subsequent regenerative processes [41].

LIF (leukemia inhibitory factor) is a cytokine with pro-regenerative and inflammation-modulating properties. It supports the angiogenic differentiation [42] of mesenchymal stem cells (MSCs). The regulation of osteogenic differentiation is more complex, LIF seems to exert a biphasic effect on it. In the early phase, osteogenic differentiation is supported, whereas in confluent, more mature MSC cultures, osteogenic differentiation is suppressed [43]. In relation to the situation in the early bone defect into which undifferentiated MSC migrate, LIF could therefore exert a more pro-osteogenic effect.

CCL17 is produced by antigen-presenting cells such as dendritic cells and macrophages, but also by endothelial cells, and is a key regulator of inflammatory and immunomodulatory processes. The CCL17 receptor CCR4 attracts Th2 cells in particular, but also NK cells and macrophages [44]. MSC also express CCR4 after TNF-α stimulation, and CCR4 is involved in the migration of MSC into damaged tissues [45].

CCL20 is broadly released by endothelial cells and macrophages that respond to stimuli such as IL-6, IFN-γ, TNF-α, IL-1β [46]. It acts via its receptor CCR6 as a chemotactic factor that attracts T- and B-lymphocytes but not monocytes [47,48]. CCL20 could therefore play a role in controlling inflammation in the early phase of bone healing. However, the receptor CCR6 is also expressed by MSC, which could influence their migration into the defect area [49].

Notably, CCL22, ICAM-1, NOV, and WISP-1 increased across all groups, indicating enhanced immune cell recruitment, angiogenic activity, and osteoprogenitor activation. Matrix remodeling was reflected by increased MMP3 and MMP9 levels. Conversely, IL-1RA decreased at day seven, consistent with resolution of acute inflammation. Together, these findings suggest a coordinated transition from inflammation toward tissue repair, with overlapping pro- and anti-regenerative signals tightly regulating osteogenesis.

### 4.4. Dynamics of the Protein Release: Factors Differentially Released by D7 Samples

In addition to these newly expressed proteins, dynamic changes in other mediators were observed. Ten factors showed significant or trending alterations between day three and day seven, with most increasing over time. In nine cases, expression increased in one or more experimental groups and remained at least at the same level in the other experimental groups. In one case, a significant decrease was observed. The extent but not the direction of the changes was often dependent on the cell loading. An increase in expression was recorded in all experimental groups for CCL22, ICAM-1, NOV and WISP-1. Other factors such as galectin-1, GDF-15, LIX, MMP3 and MMP9 increased in the median in all groups at D7, but only partially significantly. IL-1RA was downregulated at D7 in all groups, significantly for the hADM group and in trend for the hADM + BMC group. In the early phase of bone regeneration (approx. 1 week), chemotactic cytokines and adhesion molecules played an important role in the initial inflammatory reaction, cell recruitment and new tissue formation [50,51].

Therefore, an increase in these mediators indicates a coordinated phase of transition from acute inflammation to tissue repair: In the early inflammatory phase on day three, there was a strong recruitment of neutrophils and monocytes by the factors CCL22, ICAM-1 and LIX, which possibly led to a further release of inflammatory mediators and could explain the increase on day seven. The detection of MMP3 and MMP9 suggests initial extracellular matrix (ECM) remodeling. The changes on day seven indicate the transition phase to regeneration [52]. In addition, a change in anti-inflammatory mediators was observed; the decrease in IL-1RA indicates a decrease in acute inflammation, carried forward by the first line cytokine IL-1β. The increase in galectin-1 indicates the regulation of secondary inflammatory processes. Galectin-1 has been shown to trigger apoptosis of T cells. It also promotes the differentiation of naïve immune cells to a suppressive phenotype, the recruitment of immunosuppressive cells, and can dampen the activity of cytotoxic leukocytes [53].

The increased expression of NOV and WISP-1 indicates increased angiogenic and osteogenic activity (NOV) and activation of osteoprogenitor cells (WISP-1). NOV is expressed during wound healing and induces angiogenesis in vivo [54]. Interestingly, it is also described that NOV is able to stimulate mouse and human skeletal stem cell activity, increase bone remodeling, and accelerate fracture repair in young and old mice of both sexes [55]. NOV binds BMP-2 and inhibits its functions in promoting osteogenic differentiation [56] by antagonizing BMP2 and Wnt-signaling [57]. WISP-1, on the other hand, promotes MSC proliferation, osteoblastic differentiation, and attenuates chondrogenic differentiation. Furthermore, WISP-1 binds BMP-2 and thus enhances its effect in the context of osteogenesis [58]. The simultaneous expression of pro- and anti-osteogenic factors may indicate tight regulation of BMP-2-mediated osteogenesis in the bone defect.

The further increase in MMP3 and MMP9 expression at day seven is indicative of ongoing ECM modeling to enable new tissue structures. Taken together, the new mediators that were released on day seven and the altered expression of other mediators on day seven suggest a regulated balance between inflammation, ECM remodeling, cell recruitment, and bone healing.

### 4.5. Impact of CD8-Depletion on Cell Differentiation

Interestingly, cell loading with BMC or CD8-depleted BMC only subtly influenced the profile of released proteins. No factor was exclusively dependent on cell loading, and most differences were relatively small.

Nevertheless, a three-day incubation with the respective conditioned media during the differentiation process was sufficient to reveal differences in the expression of relevant genes in osteogenically stimulated MSCs and macrophage subtypes. In MSCs, collagen-1α expression was consistently upregulated in all hADM groups compared with controls, independent of cell seeding. CM-CD8-7 further enhanced IGF1 expression significantly and BMP2 expression modestly, pointing to a pro-osteogenic effect of CD8-depleted BMCs. Potential mediators in conditioned media include IL-1β, which has been shown to induce IGF1 and BMP2, although no relevant group differences in IL-1β concentrations were detected, and low-dose TGF-β, which remained below the detection threshold in our assays [59]. However, NOV gene expression was also induced, which can have a proangiogenic but also osteogenesis-inhibiting effect [54,60]. Functionally, ALP activity was reduced in most groups but restored to control levels in CM-CD8-7. This suggests that only in this condition the balance of stimulatory mediators (e.g., IL-6) versus inhibitory mediators (e.g., IL-1β) may have shifted toward osteogenic stimulation [61,62]. Taken together, the results can be interpreted in the direction of a slight preference of pro-regenerative and pro-osteogenic processes by medium conditioned with CM-CD8-7, an assumption that is also supported by the mediator profile in the conditioned medium of D7 samples.

### 4.6. Differentiation of Macrophage Subtypes

The conditioned media exerted distinct effects on the expression of canonical M1 and M2 macrophage marker genes. In particular, M0 macrophages exhibited marked changes in the expression of the M1 marker iNOS as well as the M2 markers Arginase-1 and SOCS-1 following exposure to CM-hADM-7.

Cytokines known to induce iNOS and detectable in the CMs included IL-1α and IL-1β [63]. While IL-1β concentrations were comparable across all CMs, IL-1α levels were markedly elevated in CM-hADM-7. This elevation may account for the pronounced induction of iNOS in M0 macrophages and the high median iNOS expression observed in M1-differentiated macrophages.

Arginase-1, a marker of alternatively activated (M2) macrophages, is predominantly induced by anti-inflammatory and tissue-repair signals mediated through STAT6 (IL-4/IL-13), PPARγ, or cAMP/CREB-dependent pathways [64]. Although IL-4 and IL-13 were measurable targets of the protein array, neither cytokine was detected in the CMs. The factors responsible for the pronounced Arginase-1 induction in M0 macrophages treated with CM-hADM-7, as well as for the enhanced Arginase-1 expression in M2 macrophages exposed to CM-hADM or CM-BMC, therefore remain to be identified.

The M2-associated regulatory molecule SOCS-1 was significantly upregulated in M0 macrophages exposed to CM-hADM-3 and CM-BMC-7. Among known inducers, only IL-6 [64] was detectable in D7 CM, whereas IFN-γ remained below the detection threshold. Other potential regulators such as IL-10 and TGF-β [64] were not included in the protein array. Consequently, based on the current dataset, the mechanisms driving SOCS-1 induction in M0 macrophages cannot yet be fully elucidated.

### 4.7. Macrophage Response to Conditioned Media: Regulation of CCL2 and IL-1RA Expression

In addition to lineage marker genes, the gene expression of the inflammatory mediator CCL2 and the anti-inflammatory regulator IL-1RA was analyzed in M0-, M1-, and M2-macrophages in response to the different CMs.

CCL2 is known to be induced in macrophages by IFN-γ, IL-1α/β, LPS, and TNF-α [65]. Since IFN-γ and TNF-α were not detectable in the CMs, it is likely that IL-1, particularly IL-1α, contributed predominantly to the observed CCL2 induction. The highest IL-1α concentrations were measured in CM-hADM-3, which may explain the strong upregulation of CCL2 following incubation with this CM. In contrast, the low CCL2 expression observed after incubation with CM-hADM-CD8 from D3 and D7 suggests that the composition of these media creates a more anti-inflammatory environment, potentially favorable for bone healing.

IL-1RA, a natural antagonist of the IL-1 receptor that blocks IL-1α/β signaling without receptor activation [66], was consistently induced by all CMs across the macrophage subtypes analyzed. This upregulation may represent a counter-regulatory mechanism balancing elevated IL-1 levels, particularly in CM-hADM-3, and highlights the macrophage’s role in controlling and resolving inflammation.

Finally, this experiment underscores that the type of cellular loading of the membranes markedly influences the biological activity of their conditioned media. Compared to protein array analyses, which revealed only minor compositional differences between CMs, this functional macrophage assay appears more sensitive and informative in reflecting the biological impact of the membranes with their specific cellular loads.

### 4.8. Limitations

The main limitation of this study is the small group size (*n* = 3), which reduces statistical power. In addition, we report exploratory rather than confirmatory significances, as no alpha correction for multiple testing was applied. Therefore, the findings should be interpreted as hypothesis-generating and require validation in larger studies.

The proteome profiler detects only a predefined set of 79 proteins, leaving parts of the secretome unmapped. In addition, certain proteins from this set may have been expressed but were below the detection limit of the proteome profiler. The assay is semi-quantitative, and due to limited conditioned medium volumes, results could not be validated by quantitative assays such as ELISA.

## 5. Conclusions

The duration of hADM presence in vivo significantly shapes the protein signature, with a shift from inflammation to regeneration observed after seven days. On day seven, protein release indicated a shift from inflammation to regeneration, possibly involving key peptides such as RGM-A, osteoprotegerin, LIF, IL-6, CCL20 and CCL17, which play an interlinked role in inflammation, cell recruitment, and bone remodeling. While differences between BMC and CD8-depleted BMC loading of hADMs were modest with regard to protein release, CD8-depletion favored a pro-regenerative environment, as reflected in MSC osteogenic gene expression and macrophage polarization. These findings support the concept of hADM as a dynamic bioreactor that acquires biological activity through host cell infiltration, paralleling the Masquelet-induced membrane.

## Figures and Tables

**Table 1:** Abbreviation for CM preparations. Differentially pretreated hADM were wrapped around bone defects filled with syngenic spongiosa and removed after 3 or 7 days in situ. For conditioning, medium was incubated with the explanted hADM for 24 h. The concentration of CM was 10% in all subsequent experiments.

Designation	Treatment	Time In Situ (d)
CM-hADM-3	hADM without BMC	3
CM-hADM-7	hADM without BMC	7
CM-BMC-3	hADM loaded with BMC	3
CM-BMC-7	hADM loaded with BMC	7
CM-CD8-3	hADM loaded with BMC-CD8	3
CM-CD8-7	hADM loaded with BMC-CD8	7

**Table 2:** Genes, corresponding protein names, and primers used for gene expression analysis in MSC and macrophages.

Analyzed Gene (MSC)	Protein	GeneGlobe ID:
*Bmp2*	BMP-2	PPM03753A
*Col1a1*	Collagen1α	PPR42922A
*Igf1*	IGF-1	PPR06664F
*Ccn3*	Nov	PPR48283A
**Analyzed gene (macrophages)**		**GeneGlobe ID:**
*Arg1*	Arginase	PPR44521A
*Ccl2*	CCL-2	PPR06714B
*Il1rn*	IL-1RA	PPR06438A
*Nos2*	iNOS	PPR75758A
*Socs1*	SOCS-1	PPR52395A
**Analyzed gene (housekeeping)**		**GeneGlobe ID:**
*Gapdh*	GAPDH	PPR06557B

## Data Availability

The original contributions presented in this study are included in the article. Further inquiries can be directed to the corresponding author.

## Supplementary Materials

The following supporting information can be downloaded at: https://www.mdpi.com/article/10.3390/cells15030215/s1, Table S1: Proteins secreted from hADMs retrieved 3 or 7 days postoperatively.

## Abbreviations

The following abbreviations are used in this manuscript:

PMMA	Polymethylmethacrylate
hADM	Human acellular dermal matrix
BMC	Bone marrow mononuclear cells
IFN-γ	Interferon gamma
TNF-α	Tumor necrosis factor alpha
G	Group
MSC	Mesenchymal stem cells
PBS	Phosphate-buffered saline
CM	Conditioned medium
GM	Growth medium
IL	Interleukin

## References

[1] Durand M., Mathieu L., Venant J., Masquelet A.-C., Collombet J.-M. (2025). Engineering the bone reconstruction surgery: The case of the masquelet-induced membrane technique. Eur. J. Trauma Emerg. Surg..

[2] Verboket R.D., Leiblein M., Janko M., Schaible A., Brune J.C., Schröder K., Heilani M., Fremdling C., Busche Y., Irrle T. (2020). From two stages to one: Acceleration of the induced membrane (Masquelet) technique using human acellular dermis for the treatment of non-infectious large bone defects. Eur. J. Trauma Emerg. Surg..

[3] Verboket R.D., Henrich D., Janko M., Sommer K., Neijhoft J., Söhling N., Weber B., Frank J., Marzi I., Nau C. (2024). Human Acellular Collagen Matrices-Clinical Opportunities in Tissue Replacement. Int. J. Mol. Sci..

[4] Seebach C., Henrich D., Schaible A., Relja B., Jugold M., Bönig H., Marzi I. (2015). Cell-based therapy by implanted human bone marrow-derived mononuclear cells improved bone healing of large bone defects in rats. Tissue Eng. Part A.

[5] Janko M., Pöllinger S., Schaible A., Bellen M., Schröder K., Heilani M., Fremdling C., Marzi I., Nau C., Henrich D. (2020). Determination of the effective dose of bone marrow mononuclear cell therapy for bone healing in vivo. Eur. J. Trauma Emerg. Surg..

[6] Leiblein M., Kolb T., Christian L., Schröder K., Yaman C., Schaible A., Marzi I., Henrich D., Janko M. (2020). Introduction of a New Surgical Method to Improve Bone Healing in a Large Bone Defect by Replacement of the Induced Membrane by a Human Decellularized Dermis Repopulated with Bone Marrow Mononuclear Cells in Rat. Materials.

[7] Penna-Martinez M., Kammerer A., Stützle P., Fees S., Behr S., Schaible I., Schröder K., Verboket R.D., Neijhoft J., Marzi I. (2024). Enhancement of a one-step membrane technique for the treatment of large bone defects by pre-seeding the membrane with CD8 lymphocyte depleted bone marrow mononuclear cells in a rat femoral defect model. Front. Immunol..

[8] Reinke S., Geissler S., Taylor W.R., Schmidt-Bleek K., Juelke K., Schwachmeyer V., Dahne M., Hartwig T., Akyüz L., Meisel C. (2013). Terminally differentiated CD8^+^ T cells negatively affect bone regeneration in humans. Sci. Transl. Med..

[9] Ortona E., Pagano M.T., Capossela L., Malorni W. (2023). The Role of Sex Differences in Bone Health and Healing. Biology.

[10] Wang H., Li Y., Li H., Yan X., Jiang Z., Feng L., Hu W., Fan Y., Lin S., Li G. (2025). T cell related osteoimmunology in fracture healing: Potential targets for augmenting bone regeneration. J. Orthop. Transl..

[11] Rössner E., Smith M.D., Petschke B., Schmidt K., Vitacolonna M., Syring C., von Versen R., Hohenberger P. (2011). Epiflex(®) a new decellularised human skin tissue transplant: Manufacture and properties. Cell Tissue Bank..

[12] Verboket R.D., Söhling N., Heilani M., Fremdling C., Schaible A., Schröder K., Brune J.C., Marzi I., Henrich D. (2022). The Induced Membrane Technique-The Filling Matters: Evaluation of Different Forms of Membrane Filling with and without Bone Marrow Mononuclear Cells (BMC) in Large Femoral Bone Defects in Rats. Biomedicines.

[13] Söhling N., Heilani M., Fremdling C., Schaible A., Schröder K., Brune J.C., Eras V., Nau C., Marzi I., Henrich D. (2023). One Stage Masquelets Technique: Evaluation of Different Forms of Membrane Filling with and without Bone Marrow Mononuclear Cells (BMC) in Large Femoral Bone Defects in Rats. Cells.

[14] Söhling N., Ondreka M., Kontradowitz K., Reichel T., Marzi I., Henrich D. (2022). Early Immune Response in Foreign Body Reaction Is Implant/Material Specific. Materials.

[15] Eldesoqi K., Henrich D., El-Kady A.M., Arbid M.S., Abd El-Hady B.M., Marzi I., Seebach C. (2014). Safety evaluation of a bioglass-polylactic acid composite scaffold seeded with progenitor cells in a rat skull critical-size bone defect. PLoS ONE.

[16] Nau C., Simon S., Schaible A., Seebach C., Schröder K., Marzi I., Henrich D. (2018). Influence of the induced membrane filled with syngeneic bone and regenerative cells on bone healing in a critical size defect model of the rat’s femur. Injury.

[17] Bianconi S., Oliveira K.M.C., Klein K.-L., Wolf J., Schaible A., Schröder K., Barker J., Marzi I., Leppik L., Henrich D. (2023). Pretreatment of Mesenchymal Stem Cells with Electrical Stimulation as a Strategy to Improve Bone Tissue Engineering Outcomes. Cells.

[18] Jin X., Li Y., Chen X., Chen J., Xu J. (2022). Isolation of Monocyte-Macrophage Lineage Cells from Rat Bones by Secondary Adherence Method. J. Vis. Exp..

[19] Guo X.-Y., Wang S.-N., Wu Y., Lin Y.-H., Tang J., Ding S.-Q., Shen L., Wang R., Hu J.-G., Lü H.-Z. (2019). Transcriptome profile of rat genes in bone marrow-derived macrophages at different activation statuses by RNA-sequencing. Genomics.

[20] Bender R., Lange S. (2001). Adjusting for multiple testing—When and how?. J. Clin. Epidemiol..

[21] Rothman K.J. (1990). No adjustments are needed for multiple comparisons. Epidemiology.

[22] Fonseka P., Pathan M., Chitti S.V., Kang T., Mathivanan S. (2021). FunRich enables enrichment analysis of OMICs datasets. J. Mol. Biol..

[23] Gessmann J., Rosteius T., Baecker H., Sivalingam K., Peter E., Schildhauer T.A., Köller M. (2022). Is the bioactivity of induced membranes time dependent?. Eur. J. Trauma Emerg. Surg..

[24] Gindraux F., Loisel F., Bourgeois M., Oudina K., Melin M., de Billy B., Sergent P., Leclerc G., Petite H., Auber F. (2020). Induced membrane maintains its osteogenic properties even when the second stage of Masquelet’s technique is performed later. Eur. J. Trauma Emerg. Surg..

[25] Masquelet A.C., Begue T. (2010). The concept of induced membrane for reconstruction of long bone defects. Orthop. Clin. N. Am..

[26] Nau C., Henrich D., Seebach C., Schröder K., Fitzsimmons S.-J., Hankel S., Barker J.H., Marzi I., Frank J. (2016). Treatment of Large Bone Defects with a Vascularized Periosteal Flap in Combination with Biodegradable Scaffold Seeded with Bone Marrow-Derived Mononuclear Cells: An Experimental Study in Rats. Tissue Eng. Part A.

[27] Seebach C., Nau C., Henrich D., Verboket R., Bellen M., Frischknecht N., Moeck V., Eichler K., Horlohé K.H.S., Hoffmann R. (2024). Cell-Based Therapy by Autologous Bone Marrow-Derived Mononuclear Cells for Bone Augmentation of Plate-Stabilized Proximal Humeral Fractures: A Multicentric, Randomized, Open Phase IIa study. Stem Cells Transl. Med..

[28] Seebach C., Henrich D., Meier S., Nau C., Bonig H., Marzi I. (2016). Safety and feasibility of cell-based therapy of autologous bone marrow-derived mononuclear cells in plate-stabilized proximal humeral fractures in humans. J. Transl. Med..

[29] Elgaz S., Kuçi Z., Kuçi S., Bönig H., Bader P. (2019). Clinical Use of Mesenchymal Stromal Cells in the Treatment of Acute Graft-versus-Host Disease. Transfus. Med. Hemotherapy.

[30] Leppik L., Gempp A., Kuçi Z., Kuçi S., Bader P., Bönig H., Marzi I., Henrich D. (2022). A New Perspective for Bone Tissue Engineering: Human Mesenchymal Stromal Cells Well-Survive Cryopreservation on β-TCP Scaffold and Show Increased Ability for Osteogenic Differentiation. Int. J. Mol. Sci..

[31] Bonig H., Verbeek M., Herhaus P., Braitsch K., Beutel G., Schmid C., Müller N., Bug G., Döring M., von Stackelberg A. (2023). Real-world data suggest effectiveness of the allogeneic mesenchymal stromal cells preparation MSC-FFM in ruxolitinib-refractory acute graft-versus-host disease. J. Transl. Med..

[32] Bernemann I., Mueller T., Blasczyk R., Glasmacher B., Hofmann N. (2011). Colonization of collagen scaffolds by adipocytes derived from mesenchymal stem cells of the common marmoset monkey. Biochem. Biophys. Res. Commun..

[33] Duffy M.M., Ritter T., Ceredig R., Griffin M.D. (2011). Mesenchymal stem cell effects on T-cell effector pathways. Stem Cell Res. Ther..

[34] Akhir H.M., Teoh P.L. (2020). Collagen type I promotes osteogenic differentiation of amniotic membrane-derived mesenchymal stromal cells in basal and induction media. Biosci. Rep..

[35] Advani D., Farid N., Tariq M.H., Kohli N. (2025). A systematic review of mesenchymal stem cell secretome: Functional annotations, gene clusters and proteomics analyses for bone formation. Bone.

[36] Corradini E., Babitt J.L., Lin H.Y. (2009). The RGM/DRAGON family of BMP co-receptors. Cytokine Growth Factor Rev..

[37] Wu Q., Sun C.C., Lin H.Y., Babitt J.L. (2012). Repulsive guidance molecule (RGM) family proteins exhibit differential binding kinetics for bone morphogenetic proteins (BMPs). PLoS ONE.

[38] Mirakaj V., Brown S., Laucher S., Steinl C., Klein G., Köhler D., Skutella T., Meisel C., Brommer B., Rosenberger P. (2011). Repulsive guidance molecule-A (RGM-A) inhibits leukocyte migration and mitigates inflammation. Proc. Natl. Acad. Sci. USA.

[39] Kon T., Cho T.J., Aizawa T., Yamazaki M., Nooh N., Graves D., Gerstenfeld L.C., Einhorn T.A. (2001). Expression of osteoprotegerin, receptor activator of NF-kappaB ligand (osteoprotegerin ligand) and related proinflammatory cytokines during fracture healing. J. Bone Miner. Res..

[40] Tong X., Gu J., Song R., Wang D., Sun Z., Sui C., Zhang C., Liu X., Bian J., Liu Z. (2019). Osteoprotegerin inhibit osteoclast differentiation and bone resorption by enhancing autophagy via AMPK/mTOR/p70S6K signaling pathway in vitro. J. Cell. Biochem..

[41] Prystaz K., Kaiser K., Kovtun A., Haffner-Luntzer M., Fischer V., Rapp A.E., Liedert A., Strauss G., Waetzig G.H., Rose-John S. (2018). Distinct Effects of IL-6 Classic and Trans-Signaling in Bone Fracture Healing. Am. J. Pathol..

[42] Santos G.C., Silva D.N., Fortuna V., Silveira B.M., Orge I.D., de Santana T.A., Sampaio G.L., Paredes B.D., Ribeiro-Dos-Santos R., Soares M.B.P. (2020). Leukemia Inhibitory Factor (LIF) Overexpression Increases the Angiogenic Potential of Bone Marrow Mesenchymal Stem/Stromal Cells. Front. Cell Dev. Biol..

[43] Malaval L., Aubin J.E. (2001). Biphasic effects of leukemia inhibitory factor on osteoblastic differentiation. J. Cell. Biochem..

[44] Zou S., Liu B., Feng Y. (2024). CCL17, CCL22 and their receptor CCR4 in hematologic malignancies. Discov. Oncol..

[45] Szydlak R. (2019). Mesenchymal stem cells’ homing and cardiac tissue repair. Acta Biochim. Pol..

[46] Cui L.-Y., Chu S.-F., Chen N.-H. (2020). The role of chemokines and chemokine receptors in multiple sclerosis. Int. Immunopharmacol..

[47] Nelson R.T., Boyd J., Gladue R.P., Paradis T., Thomas R., Cunningham A.C., Lira P., Brissette W.H., Hayes L., Hames L.M. (2001). Genomic organization of the CC chemokine mip-3alpha/CCL20/larc/exodus/SCYA20, showing gene structure, splice variants, and chromosome localization. Genomics.

[48] Hieshima K., Imai T., Opdenakker G., van Damme J., Kusuda J., Tei H., Sakaki Y., Takatsuki K., Miura R., Yoshie O. (1997). Molecular cloning of a novel human CC chemokine liver and activation-regulated chemokine (LARC) expressed in liver Chemotactic activity for lymphocytes and gene localization on chromosome 2. J. Biol. Chem..

[49] Sajjad U., Ahmed M., Iqbal M.Z., Riaz M., Mustafa M., Biedermann T., Klar A.S. (2024). Exploring mesenchymal stem cells homing mechanisms and improvement strategies. Stem Cells Transl. Med..

[50] Li R., Ye J.J., Gan L., Zhang M., Sun D., Li Y., Wang T., Chang P. (2023). Traumatic inflammatory response: Pathophysiological role and clinical value of cytokines. Eur. J. Trauma Emerg. Surg..

[51] Ridiandries A., Tan J.T.M., Bursill C.A. (2018). The Role of Chemokines in Wound Healing. Int. J. Mol. Sci..

[52] Kandhwal M., Behl T., Singh S., Sharma N., Arora S., Bhatia S., Al-Harrasi A., Sachdeva M., Bungau S. (2022). Role of matrix metalloproteinase in wound healing. Am. J. Transl. Res..

[53] Yu X., Qian J., Ding L., Yin S., Zhou L., Zheng S. (2023). Galectin-1: A Traditionally Immunosuppressive Protein Displays Context-Dependent Capacities. Int. J. Mol. Sci..

[54] Lin C.G., Leu S.-J., Chen N., Tebeau C.M., Lin S.-X., Yeung C.-Y., Lau L.F. (2003). CCN3 (NOV) is a novel angiogenic regulator of the CCN protein family. J. Biol. Chem..

[55] Babey M.E., Krause W.C., Chen K., Herber C.B., Torok Z., Nikkanen J., Rodriguez R., Zhang X., Castro-Navarro F., Wang Y. (2024). A maternal brain hormone that builds bone. Nature.

[56] Minamizato T., Sakamoto K., Liu T., Kokubo H., Katsube K., Perbal B., Nakamura S., Yamaguchi A. (2007). CCN3/NOV inhibits BMP-2-induced osteoblast differentiation by interacting with BMP and Notch signaling pathways. Biochem. Biophys. Res. Commun..

[57] Rydziel S., Stadmeyer L., Zanotti S., Durant D., Smerdel-Ramoya A., Canalis E. (2007). Nephroblastoma overexpressed (Nov) inhibits osteoblastogenesis and causes osteopenia. J. Biol. Chem..

[58] Ono M., Inkson C.A., Kilts T.M., Young M.F. (2011). WISP-1/CCN4 regulates osteogenesis by enhancing BMP-2 activity. J. Bone Miner. Res..

[59] Jerde T.J., Bushman W. (2009). IL-1 induces IGF-dependent epithelial proliferation in prostate development and reactive hyperplasia. Sci. Signal..

[60] Matsushita Y., Sakamoto K., Tamamura Y., Shibata Y., Minamizato T., Kihara T., Ito M., Katsube K., Hiraoka S., Koseki H. (2013). CCN3 protein participates in bone regeneration as an inhibitory factor. J. Biol. Chem..

[61] Bastidas-Coral A.P., Hogervorst J.M.A., Forouzanfar T., Kleverlaan C.J., Koolwijk P., Klein-Nulend J., Bakker A.D. (2019). IL-6 counteracts the inhibitory effect of IL-4 on osteogenic differentiation of human adipose stem cells. J. Cell. Physiol..

[62] Voss J.O., Loebel C., Bara J.J., Fussinger M.A., Duttenhoefer F., Alini M., Stoddart M.J. (2015). Effect of Short-Term Stimulation with Interleukin-1β and Differentiation Medium on Human Mesenchymal Stromal Cell Paracrine Activity in Coculture with Osteoblasts. Biomed Res. Int..

[63] LaPointe M.C., Sitkins J.R. (1996). Mechanisms of interleukin-1beta regulation of nitric oxide synthase in cardiac myocytes. Hypertension.

[64] Sica A., Mantovani A. (2012). Macrophage plasticity and polarization: In vivo veritas. J. Clin. Investig..

[65] Deshmane S.L., Kremlev S., Amini S., Sawaya B.E. (2009). Monocyte chemoattractant protein-1 (MCP-1): An overview. J. Interf. Cytokine Res..

[66] Dinarello C.A. (2011). Interleukin-1 in the pathogenesis and treatment of inflammatory diseases. Blood.

