# Supplementary material for: Cell Supported Single Membrane Technique for the Treatment of Large Bone Defects: Depletion of CD8+ Cells Enhances Bone Healing Mechanisms During the Early Bone Healing Phase
Source: Cells. 2026 Jan 23;15(3):215. doi: 10.3390/cells15030215 (PMC12897119; doi:10.3390/cells15030215)
Supplement: Supplementary file 1 [file cells-15-00215-s001.zip › cells-4050450-supplementary.pdf]

Supplementary Table S1. Proteins secreted from hADMs retrieved 3 or 7 days postoperatively. Duration of hADM in vivo, primary functions and cellular sources of secreted proteins are listed. Proteins newly secreted from d7 hADMs are bold type. Y,n = detectable yes, no; DC=dendritic cells

#	Name	d3	d7	Function	Cellular sources
1	Adiponectin	y	y	Reduce inflammation, protect from apoptosis, enhancing insulin sensitivity	Mainly adipocytes
2	CCL (CC-chemokine ligand)-2	y	y	Recruits monocytes, macrophages, and dendritic cells to inflammation sites.	Fibroblasts, endothelial cells, macrophages
3	CCL3	y	y	Promotes inflammation, activates macrophages and T cells	Macrophages, DC, T cells.
4	**CCL17**	**n**	**y**	Recruits T helper (Th2) cells and regulates immune responses	DC, macrophages
5	**CCL20**	**n**	**y**	Attracts lymphocytes and dendritic cells	macrophages
6	CCL21	y	y	Guides T cells and dendritic cells into secondary lymphoid organs	Endothelial cells, fibroblasts
7	CCL22	y	y	Attracts T helper (Th2) cells	DC, macrophages
8	Clusterin	y	y	Protects cells, inhibits apoptosis, modulates immunity cell adhesion and tissue remodeling	Hepatocytes, epithelial cells some immune cells
9	CXCL (CX-chemokine ligand)-2	y	y	Recruits neutrophils, pro-inflammatory	Macrophages, endothelial cells
10	CXCL7	y	y	Promotes neutrophil recruitment, plasminogen activator	Platelets
11	Cystatin C	y	y	Inhibits cysteine proteases, regulates proteolysis.	Nucleated cells
12	DPP(Dipeptidylpeptidase) -IV	y	y	Regulates immune responses, glucose metabolism	Endothelial cells, T cells
13	Endostatin	y	y	Inhibits angiogenesis	Fibroblasts, endothelial cells

14	Fetuin A	y	y	Inhibits calcification, regulates insulin sensitivity, inflammation	Hepatocytes
15	Fibulin-3	y	y	Organizes extracellular matrix, cell adhesion	Fibroblasts, endothelial cells
16	Galectin-1	y	y	Immune regulation, cell adhesion	Endothelial cells, immune cells
17	Galectin-3	y	y	Promotes inflammation, cell proliferation, fibrosis	Macrophages, fibroblasts
18	G-CSF (granulocyte-colony stimulating factor)	y	y	Promotes neutrophil production and maturation	Fibroblasts, endothelial cells, macrophages, some epithelial cells
19	GDF (growth / differentiation factor)-15	y	y	Protects cells during tissue damage, modulates immunity	Macrophages, endothelial cells
20	Hepassocin<br>(Hepatocyte-derived growth factor)<br>Fibrinogen-Like Protein 1	y	y	Promotes liver regeneration, supports cell growth and liver repair processes	Hepatocytes
21	ICAM (Intercellular adhesion molecule)-1	y	y	Cell adhesion, immune cell migration	Endothelial cells, macrophages
22	IGF (Insulin like growth factor)-1	y	y	Promotes cell growth and regeneration	Hepatocytes
23	IGFBP(binding protein)-2	y	y	Regulate IGF-1 activity	hepatocytes, brain cells,<br>adipocytes, fibroblasts and endothelial cells
24	IGFBP-3	y	y	Regulate IGF-1 activity	Hepatocytes, bone<br>marrow cells and skin cells
25	IGFBP-5	y	y	Regulate IGF-1 activity	Fibroblasts, osteoblasts, and muscle cells

26	IGFBP-6	y	y	Regulate IGF-1 activity	Placental cells and fetal cells
27	IL(Interleukin)-1α	y	y	Key inflammatory mediator	Macrophages
28	IL-1β	y	y	Key inflammatory mediator	Macrophages
29	IL-1RA	y	y	Inhibits IL-1α/β-mediated inflammation	Monocytes, fibroblasts
30	**IL-6**	**n**	**y**	Regulates inflammation and immune response, s**upports cell growth and differentiation**	Macrophages, , **T cells** fibroblasts, e**ndothelial cells and** adipocytes
31	Jagged-1	y	y	Involved in Notch signaling, cell communication	Endothelial cells, fibroblasts
32	**LIF (leukemia inhibitory factor)**	**n**	**y**	Stem cell differentiation, inflammation modulation	Fibroblasts, macrophages
33	Lipocalin-2	y	y	Regulates iron homeostasis, inflammation	Neutrophils, macrophages
34	LIX	y	y	Attracts **neutrophils**, contributes to the inflammatory response and **supports tissue repair**	**Epithelial cells, endothelial cells, fibroblasts,** macrophages and neutrophils
35	MMP-2 (matrix metalloproteinase)	y	y	Breakdown of extracellular matrix	Fibroblasts, macrophages
36	MMP-3	y	y	Breakdown of extracellular matrix	Fibroblasts, macrophages
37	MMP-9	y	y	Breakdown of extracellular matrix	Fibroblasts, macrophages
38	NOV (nephoblastoma overexpressed)	y	y	Regulates cell proliferation and osteogenic differentiation	Fibroblasts
39	Osteopontin	y	y	Promotes cell migration and adhesion, bone mineralization	Osteoblasts, **macrophages, T cells, epithelial cells and fibroblasts**

40	**Osteoprotegerin**	**n**	**y**	Inhibits osteoclast activity	Osteoblasts, fibroblasts
41	RBP (Retinol binding protein)-4	y	y	Transporter for retinol	Hepatocytes, adipocytes
42	Resistin	y	y	Regulates Insulin resistance, promotes **inflammation and affects lipid metabolism**	adipocytes, macrophages and monocytes
43	**RGM (Repulsive guidance molecule)-A** BMP-Co-receptor	**n**	**y**	Controls neuronal cells, regulates cell growth and differentiation and contributes to bone and tissue regeneration	neuronal cells, glial cells and osteoblasts
44	Serpin E1	y	y	Reduces fibrinolysis, ECM remodeling	Endothelial cells, fibroblasts and **platelets**
45	VCAM (Vascular cell adhesion protein)-1	y	y	Cell adhesion, immune cell migration	Endothelial cells
46	VEGF (Vascular endothelial growth factor)	y	y	Promotes angiogenesis	Endothelial cells, fibroblasts; **macrophages** and **platelets**
47	WISP (WNT1-inducible-signaling pathway protein)-1	y	y	Promotes cell proliferation; differentiation, extracellular matrix production and wound healing	Fibroblasts, chondrocytes and smooth muscle cells
